# The continuum between semelparity and iteroparity: plastic expression of parity in response to season length manipulation in *Lobelia inflata*

**DOI:** 10.1186/1471-2148-14-90

**Published:** 2014-04-26

**Authors:** P William Hughes, Andrew M Simons

**Affiliations:** 1 Department of Biology, Carleton University, 1125 Colonel By Drive, Ottawa K1S 5B6, Canada

**Keywords:** Semelparity, Iteroparity, Life-history theory, Reproductive effort, Phenotypic plasticity

## Abstract

**Background:**

Semelparity and iteroparity are considered to be distinct and alternative life-history strategies, where semelparity is characterized by a single, fatal reproductive episode, and iteroparity by repeated reproduction throughout life. However, semelparous organisms do not reproduce instantaneously; typically reproduction occurs over an extended time period. If variation in reproductive allocation exists within such a prolonged reproductive episode, semelparity may be considered iteroparity over a shorter time scale.

This continuity hypothesis predicts that “semelparous” organisms with relatively low probability of survival after age at first reproduction will exhibit more extreme semelparity than those with high probability of adult survival. This contrasts with the conception of semelparity as a distinct reproductive strategy expressing a discrete, single, bout of reproduction, where reproductive phenotype is expected to be relatively invariant. Here, we manipulate expected season length—and thus expected adult survival—to ask whether *Lobelia inflata,* a classic “semelparous” plant, exhibits plasticity along a semelparous-iteroparous continuum.

**Results:**

Groups of replicated genotypes were manipulated to initiate reproduction at different points in the growing season in each of three years. In lab and field populations alike, the norm of reaction in parity across a season was as predicted by the continuity hypothesis: as individuals bolted later, they showed shorter time to, and smaller size at first reproduction, and multiplied their reproductive organs through branching, thus producing offspring more simultaneously.

**Conclusions:**

This work demonstrates that reproductive effort occurs along a semelparous-iteroparous continuum within a “semelparous” organism, and that variation in parity occurs within populations as a result of phenotypic plasticity.

## Background

The evolution of the age schedule of reproduction is of central concern to life-history theory. Organisms may be categorized according to their reproductive schedules: semelparous organisms (e.g. octopus, Pacific salmon) have a single, “big-bang” fatal reproductive episode, whereas iteroparous organisms (e.g. humans, Atlantic salmon) are capable of multiple reproductive episodes per lifetime [1-4]. Cole [5] presented a formal comparison of the fitness consequences of semelparity and iteroparity. He famously identified the persistence of iteroparity as a paradox: why is it so common, given that: (1) survival from one reproductive episode to the next is costly; and (2) to be fitness-equivalent to an iteroparous strategy, a semelparous strategy need only produce one additional offspring (to replace the parent)? This problem was first resolved by pointing out that low juvenile establishment or survival lent the iteroparous strategy a fitness advantage [6]. This model, which focused on age-specific mortality, has since been refined [7,8], but remains a general framework to explain the evolution of semelparity and iteroparity as discrete strategies.

As has been noted by others [9], defining semelparity as instantaneous and fatal reproduction is ambiguous. It is unclear whether “fatal” precludes prolonged senescence, and whether “instantaneous” reproduction precludes production of more than a single offspring. Unless only a single offspring is produced, total reproductive effort is by necessity packaged in multiple offspring that are rarely produced simultaneously. Many examples of within-individual variation in the timing of semelparous reproduction in nature have been recognized. Although some semelparous species reproduce relatively rapidly, especially long-lived semelparous plants [10], others are reproductively active for an extended period of time [4,11-14]. For example, semelparous species such as capelin and crab spiders are capable of facultative iteroparity [13,15] and many cephalopods, although considered semelparous, exhibit lengthy postreproductive senescence, with some capable of a second bout of reproduction [16]. Although each of these life histories is semelparous in the sense that there is normally a terminal reproductive episode, they do not reproduce in a “single, massive, fatal reproductive episode” [17], but distribute their total reproductive effort in multiple offspring over time.

Attempts to model the fitness effects of reproductive strategies have often compared intrinsic rates of increase of annuals and perennials, where annuals are considered to be semelparous and perennials to be iteroparous. However, many semelparous organisms, such as bamboo, cicadas, *Yucca* spp., are not annual [10]. Multivoltine insects lay more than one brood per year, and their classification as semelparous or iteroparous may depend on the time scale of reference [18]. In seasonal climates, the digitizing effect of the cost of adult survival through an especially harsh event (winter, dry season) results not only in dramatic integer changes in voltinism [19] but also in annual and perennial life histories and the illusion of a strict dichotomy between semelparity and iteroparity. Thus, categorization of life histories into semelparous and iteroparous is useful, but does not fully reflect an underlying biological reality.

We ask whether prolonged reproduction in species considered to be semelparous, although within a short lifespan, may be treated as iteroparous strategic packaging of reproductive effort in multiple bouts throughout life. Together with evidence that semelparity is evolutionarily labile, with closely related species exhibiting both semelparous and iteroparous life histories [20], it seems reasonable to consider parity as a continuum of intermediate strategies between endpoints of pure semelparity (“uniparity”, sensu Kirkendall and Stenseth [9]) and pure iteroparity, a large number of small clutches, produced in discrete reproductive bouts. Following this logic, prolonged semelparity refers to a strategy where reproduction is expressed over a longer period of time than under pure semelparity.

Although numerous examples of prolonged semelparity exist, no study has attempted to address the life-history question of whether this phenomenon is indicative of phenotypic continuity between semelparity and iteroparity [21]. Two alternative explanations for prolonged semelparity exist: it may be a single reproductive episode that simply cannot be expressed instantaneously, in which case there would be no reason to consider the phenomenon to be iteroparity. In contrast, we hypothesize that prolonged semelparity is iteroparity on a short time scale, in which case reproductive allocation within a lifetime will vary depending on expected adult survival. Differences in parity among semelparous species may exist, but it is not obvious how the continuum hypothesis could be tested in a species comparison. However, within a species, variation in reproduction along the semelparity-iteroparity continuum (i.e. more or less instantaneous semelparity) may be expressed as phenotypic plasticity, i.e. the capacity for one genotype to express multiple environment-dependent phenotypes [22-24]. If a species’ reproductive allocation pattern is phenotypically plastic, manipulating season length cues should change the instantaneousness of the reproductive episode. This hypothesis has not yet been explicitly tested in either plants or animals, but anecdotal evidence in animals exists. For example, age at first reproduction influenced the amount of reproductive effort invested in offspring production and defense in Sockeye salmon, *Oncorhynchus nerka*[25-27]. Previous work has modeled the optimal timing of initiation of the single, irreversible transition to reproduction in annual plants [28-31]. However, because reproduction in these organisms is treated as a single event, no study has addressed the question of how reproductive effort is expressed or packaged following the initiation of reproduction.

In this study, we test the parity continuum hypothesis by manipulating effective season length—and thus, expected reproductive lifespan—available to replicated genetic lineages of the monocarpic plant *Lobelia inflata*. This species provides an appropriate model for five main reasons: (1) it is classically semelparous; (2) reproductive effort is realized over a extended period of time during its single growing season in nature; (3) it is obligately autogamous, and therefore genotypically-invariant lineages are readily obtained; 4) total reproductive effort can be directly assessed because reproduction is exclusively by seed, and both male and female fitness contributions are obtained in one plant; and (5) *L. inflata* has a simple acropetal flowering pattern, where fruits form sequentially along inflorescences, making it possible to track the packaging of reproductive effort.

Manipulation of effective season length was accomplished by inducing bolting in groups of experimental plants at different times (June, July, August, September) during the growing season. Progressively later bolting over the period June through September results in diminishing time available for reproduction before the onset of frosts in mid-October. If the semelparity-iteroparity continuum hypothesis is correct, late bolting should elicit a progressively more semelparous reproductive strategy; that is, late-bolting plants should exhibit a reproductive episode that trades off other aspects of reproductive success for relatively prompt and simultaneous reproduction. Specifically, we predict that, for *L. inflata*, more extreme semelparity in response to late bolting will be expressed as short time to first flowering and small size at first flowering, more synchronous flowering (through rapid development of raceme and parallel development of fruits via branching), and the production of smaller and/or fewer seeds. In contrast, if prolonged semelparity is the expression of a single strategy (the single strategy hypothesis), late bolting will result either in no change or decelerating reproduction as a direct (nonadaptive) plastic response to declining resources and deteriorating conditions toward the end of the growing season.

## Results

Over the course of this experiment, 1,509 plants from 21 genotypic lineages were tracked (Table 1). These plants represent only those that germinated on time, rooted successfully, initiated bolting during the five-day window for each bolting group, and reproduced undisturbed (field plants were subject to herbivorous attacks from grasshoppers, which were particularly severe in 2008) until the onset of senescence.

**Table 1:** Summary data table for bolting groups by year

**Year**	**Number of Plants**								
	**June**		**July**		**August**		**September**		**Total**
	**Lab**	**Field**	**Lab**	**Field**	**Lab**	**Field**	**Lab**	**Field**	
2008	54	29	48	29	54	23	52	12	301
2009	98	66	80	70	88	66	97	33	598
2010	98	64	107	68	103	51	90	29	610
								**Total**	1509

Likelihood ratio tests showed that a GLMM explained significantly more variation in the response for three traits (size at first flower, number of fruit and number of branches), whereas a GLMM and GLM explained similar proportions of the total variation in the response variable for the other four traits (days from bolting to first flower, flowering duration, seed size and seed number; Table 2). Using these models, we found significant differences among bolting months for all seven reproductive traits included in this manipulation experiment (Table 3). In general, early-bolting plants reproduced more slowly and produced fewer, larger seeds than late-bolting plants.

**Table 2:** Likelihood ratio test results for all seven dependent variables

**Dependent Variable**	**Parameters**		**Restricted Log Likelihood**		**χ**^**2**^	**Critical Value**	**DF**	**Sig p < 0.05**	**Model Used**
	**GLMM**	**GLM**	**GLMM**	**GLM**					
**Days from bolting to first flower**	22	19	9792.829	9793.545	0.716	7.82	3	ns	GLM
**Size at First Flower**	22	19	16200.799	16215.686	14.887	7.82	3	*	GLMM
**Flowering Duration**	22	19	11144.377	11145.496	1.119	7.82	3	ns	GLM
**Branch Number**	22	19	5008.693	5026.915	18.222	7.82	3	*	GLMM
**Fruit Number**	22	19	12626.805	12645.419	18.614	7.82	3	*	GLMM
**Seed Size**	14	11	−2666.507	−2666.473	0.034	7.82	3	ns	GLM
**Seed Number**	14	11	13478.815	13480.608	1.793	7.82	3	ns	GLM

**Table 3:** Means (+/- standard error) and pairwise comparisons by bolting month for all reproductive traits assessed

	**Mean (by bolting month)**				**Homogenous Subsets (p < 0.05)**			
	**Jun**	**Jul**	**Aug**	**Sept**	**Jun**	**Jul**	**Aug**	**Sept**
Time from Bolting to First Flower (days)	37.58 (+/- 0.33)	28.91 (+/- 0.34)	24.37 (+/- 0.35)	16.48 (+/- 0.44)	A	B	C	D
Height at First Flower (mm)	126.88 (+/- 3.66)	134.62 (+/- 3.69)	120.59 (+/- 3.79)	119.83 (+/- 4.41)	AB	A	B	B
Flowering Duration (days)	42.79 (+/- 0.53)	30.10 (+/- 0.53)	25.38 (+/- 0.56)	29.11 (+/- 0.69)	A	B	C	B
Number of Branches	1.13 (+/- 0.08)	1.54 (+/- 0.08)	2.14 (+/- 0.08)	2.90 (+/- 0.10)	A	B	C	D
Number of Fruit	30.58 (+/- 1.09)	38.59 (+/- 1.10)	35.23 (+/- 1.13)	43.89 (+/- 1.32)	A	B	C	D
Seed Size (mm)	.318 (+/- .002)	N/A	N/A	.276 (+/- .003)	A	N/A	N/A	B
Seed Number	7920.51 (+/- 162.7)	N/A	N/A	9221.42 (+/- 213.9)	A	N/A	N/A	B

Homogenous subsets indicate pairwise differences assessed using Tukey tests at alpha = 0.05; levels that do not share a letter are significantly different.

**Figure 1:**
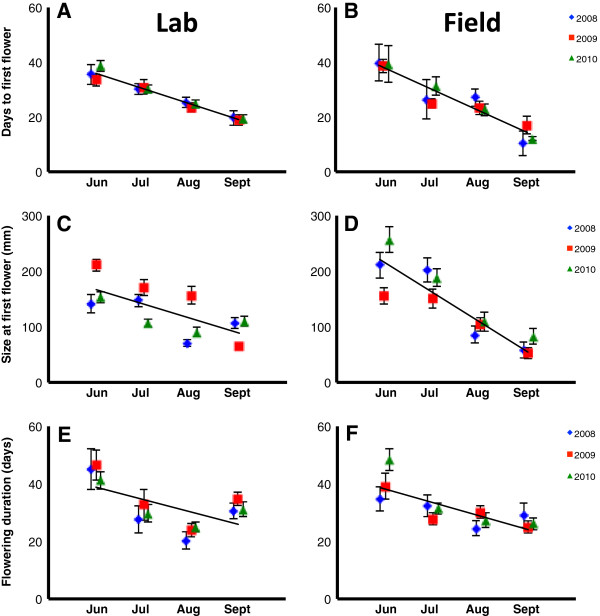
**Phenological traits by bolting date over three years.** Days to first flower **(charts A and B)** indicates the time elapsed between bolting and the formation of the first flower. Size at first flower **(charts C and D)** denotes the length of the main flowering stalk on the day the first flower is produced. Flowering duration **(charts E and F)** indicates the time elapsed between the formation of the first flower and the maturation of the last flower. Charts on the left **(A, C, E)** show lab results and those on the right **(B, D, F)** show field results.

Plasticity in the expression of all three phenological traits—time from bolting to first flower, height at first flower, and total flowering duration—was observed across manipulated bolting dates (Table 3). A GLM showed a significant effect of bolting month on days from bolting to first flower (Table 4); as plants bolted later in the season, they initiated flowering earlier (Figure 1A,B). All other predictors except year * environment were also significant for this trait. A GLMM showed a significant effect of bolting month on height at first flower (Table 5). As plants bolted later in the season, they initiated flowering at a smaller size (Figure 1C,D). Other significant predictors of size at first flowering included year * environment, bolting month * environment and year * bolting month (Table 5). A GLM showed a significant effect of bolting month on flowering duration (Table 6). Late-bolting plants flowered sooner after bolting than early-bolting plants, and the total length of time spent reproducing was significantly shorter (Figure 1E,F).

**Table 4:** Test of fixed effects from a GLM used to predict days from bolting to first flower

**Source**	**df**	**F**	**p**	**Partial η**^**2**^
Intercept	1, 1491	20616.23	< 0.0005**	0.933
Year	2, 1491	6.50	0.002*	0.009
Bolting Month	3, 1491	540.74	< 0.0005**	0.521
Environment	1, 1491	11.49	0.001*	0.008
Year * Environment	2, 1491	0.53	0.589	0.001
Bolting Month * Environment	3, 1491	23.20	< 0.0005**	0.045
Year * Bolting Month	6, 1491	4.46	< 0.0005**	0.008

*p < 0.05, **p < 0.001.

**Table 5:** Test of fixed effects from a GLMM (fitted using REML-based approximation) used to predict size (stem height) at first flower

**Source**	**df**	**F**	**p**
Intercept	1, 56.58	3214.48	< 0.0005**
Year	2, 1445.08	0.003	0.997
Bolting Month	3, 65.77	3.52	0.020*
Environment	1, 43.46	0.70	0.406
Year * Environment	2, 1350.08	69.27	< 0.0005**
Bolting Month * Environment	3, 1481.45	107.54	< 0.0005**
Year * Bolting Month	6, 1416.41	18.08	< 0.0005**

*p < 0.05, **p < 0.001.

**Table 6:** Test of fixed effects from a GLM used to predict flowering duration

**Source**	**df**	**F**	**p**	**Partial η**^**2**^
Intercept	1, 1491	11726.30	< 0.0005**	0.887
Year	2, 1491	5.98	0.003**	0.008
Bolting Month	3, 1491	196.88	< 0.0005**	0.284
Environment	1, 1491	3.88	0.049*	0.003
Year * Environment	2, 1491	11.70	< 0.0005**	0.015
Bolting Month * Environment	3, 1491	14.83	< 0.0005**	0.029
Year * Bolting Month	6, 1491	0.98	0.437	0.004

*p < 0.05, **p < 0.001.

**Figure 2:**
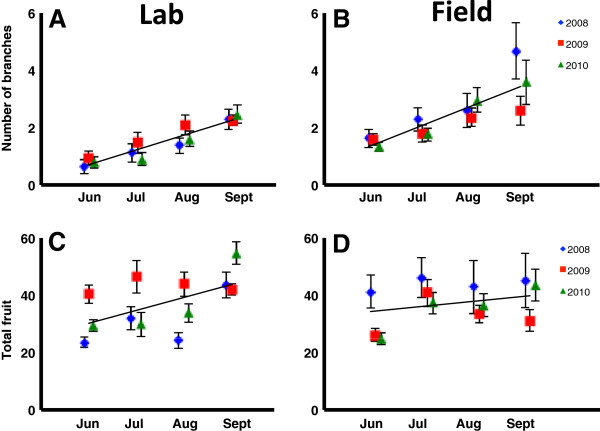
**Fruiting traits by bolting date over three years.** Number of branches **(charts A and B)** represents the mean number of branches per plant by bolting month. Total fruit number **(charts C and D)** represents the mean number of fruits per plant by bolting month. Charts on the left **(A and C)** show lab results and those on the right **(B and D)** show field results.

Plasticity was observed across manipulated bolting dates in the expression of all fruiting traits considered in this study (Table 3). A GLMM for mean number of branches showed a highly significant effect of bolting month on number of branches (Table 7). Late-bolting plants produced a greater number of branches (Figure 2A,B). Other significant predictors of number of branches included environment, year * environment, and year * bolting month. A GLMM for total number of fruit showed a highly significant effect of bolting month on number of fruit (Table 8). As plants bolted later in the season, they produced more fruit (Figure 2C,D). All interaction terms included in the GLMM were also significant predictors of number of fruit.

**Table 7:** Test of fixed effects from a GLMM (fitted using REML-based approximation) used to predict number of branches

**Source**	**df**	**F**	**p**
Intercept	1, 26.81	1237.98	< 0.0005**
Year	2, 1392.70	0.90	0.406
Bolting Month	3, 1475.18	98.38	< 0.0005**
Environment	1, 19.39	92.46	< 0.0005**
Year * Environment	2, 1223.24	11.30	< 0.0005**
Bolting Month * Environment	3, 1479.68	1.22	0.300
Year * Bolting Month	6, 1477.21	3.94	0.001**

*p < 0.05, **p < 0.001.

**Table 8:** Test of fixed effects from a GLMM (fitted using REML-based approximation) used to predict number of fruit

**Source**	**df**	**F**	**p**
Intercept	1, 53.87	2867.38	< 0.0005**
Year	2, 1447.87	0.68	0.507
Bolting Month	3, 77.41	25.49	< 0.0005**
Environment	1, 44.03	0.58	0.452
Year * Environment	2, 1390.47	46.93	< 0.0005**
Bolting Month * Environment	3, 1479.94	8.11	< 0.0005**
Year * Bolting Month	6, 1410.42	16.54	< 0.0005**

*p < 0.05, **p < 0.001.

**Figure 3:**
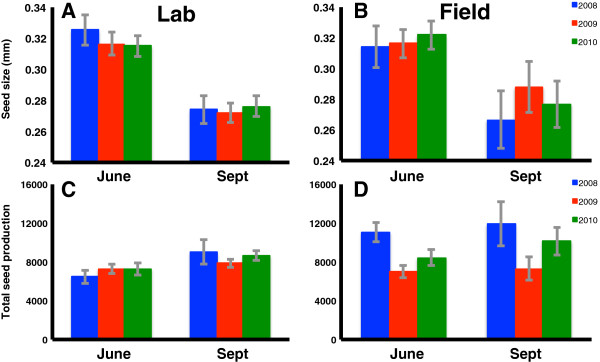
**Offspring (seed) traits by bolting date over three years.** Seed size **(charts A and B)** indicate mean seed length by bolting month (data available only for June and September bolting months). Total seed output **(charts C and D)** indicates mean number of seeds produced per plant by bolting month. Charts on the left **(A and C)** show lab results and those on the right **(B and D)** show field results.

Both seed traits analyzed in this study showed phenotypic plasticity across bolting dates (Table 3). A GLM showed a significant effect of bolting month on seed size (Table 9); late-bolting plants produced smaller seeds than early-bolting plants (Figure 3A,B). No other predictors were significant. A GLM showed a significant effect of bolting month on seed number (Table 10). Late-bolting plants produced significantly more seeds than early-bolting plants (Figure 3C,D). Other significant predictors of seed number included: year, environment, year * environment and year * bolting month.

**Table 9:** Test of fixed effects from a GLM used to predict seed size

**Source**	**df**	**F**	**p**	**Partial η**^**2**^
Intercept	1, 712	33930.27	< 0.0005**	0.979
Year	2, 712	0.29	0.747	0.001
Bolting Month	1, 712	178.86	< 0.0005**	0.201
Environment	1, 712	0.09	0.771	0.001
Year * Environment	2, 712	2.01	0.135	0.006
Bolting Month * Environment	1, 712	0.71	0.401	0.001
Year * Bolting Month	2, 712	1.12	0.327	0.001

*p < 0.05, **p < 0.001.

**Table 10:** Test of fixed effects from a GLM used to predict seed number

**Source**	**df**	**F**	**p**	**Partial η**^**2**^
Intercept	1, 712	3999.55	< 0.0005**	0.849
Year	2, 712	25.43	< 0.0005**	0.067
Bolting Month	1, 712	23.84	< 0.0005**	0.032
Environment	1, 712	34.33	< 0.0005**	0.046
Year * Environment	2, 712	20.94	< 0.0005**	0.056
Bolting Month * Environment	1, 712	0.33	0.566	0.001
Year * Bolting Month	2, 712	3.87	0.021*	0.011

*p < 0.05, **p < 0.001.

## Discussion

Reproduction in *L. inflata* showed significant phenotypic plasticity of key reproductive traits diagnostic of semelparity in response to time constraints imposed by the manipulation of bolting date. Variation in the expression of the degree of semelparity was consistent with the continuum hypothesis and inconsistent with the single strategy hypothesis: in lab and field environments alike, reproductive traits varied continuously as plants bolted later on in the season. June-bolting plants expressed prolonged semelparity (where reproductive effort was realized slowly), while September-bolting plants expressed a more instantaneous or extreme semelparity (where reproductive effort was realized quickly). Although there was substantial variation among individual plants that bolted at different times throughout the season, there were no consistent differences among the 21 genetic lineages.

Our results support the continuum hypothesis for each of our main predictions with respect to flowering traits. Perhaps most importantly, late-bolters initiated reproduction sooner after bolting and at a smaller size than did early-bolters. Flowering soon after bolting and flowering at a small size allows a plant to reproduce sooner, but may cause plants to forego fitness gains associated with production of a larger stalk, which can hold more fruit and disperses seeds farther [32]. That late-bolting plants would trade off such gains for the ability to initiate reproduction sooner and at a smaller size is consistent with the general prediction of the continuum hypothesis that late-bolting plants respond to a constrained reproductive season by adopting a more extreme semelparous reproductive strategy. Late-bolters also flowered more synchronously, by fruiting in parallel more frequently and producing many more fruit than early bolters. Although producing many flowers simultaneously may increase maximum fecundity, competition for resources between them may eventually lead to diminishing fitness gains for additional flowers [33,34].

Branching and fruiting patterns were phenotypically plastic across bolting groups. Late-bolting plants produced significantly more fruit and more branches than early-bolting plants. This pattern was not necessarily predicted by the continuum hypothesis, but it makes sense in view of the morphology of our study species: producing fruit on multiple branches allowed late-bolting plants to overcome constraints on fruit production related to the growth of the meristem; late-bolters were able to produce flowering in parallel rather than serially along the main stalk. Greater numbers of fruit also helped late-bolters produce a greater number of seeds–although early-bolters produced larger seeds, the total fecundity of late-bolters was significantly higher than that of early-bolters. Presumably, there is a context-dependent fitness cost associated with branching in *L. inflata*; otherwise, early-bolting individuals should also express branching architecture. We speculate that advantages of main stem dominance in early bolters may include better dispersal in taller plants, and higher diversification in timing of seed production, and thus in timing of germination [35]. For time-constrained plants late in the season, however, branching provides an outlet for reproductive potential that would otherwise be wasted.

Early-bolters produced fewer, larger seeds than late-bolters, a pattern that is consistent with the prediction that late-bolters would express “pure” semelparity, while early-bolters might express a more iteroparous-like semelparity [36]. Larger seeds show reduced dormancy [37], and are more likely than smaller seeds to germinate and establish rosettes within the same season. In contrast, late-bolters produced seeds in many fruits simultaneously, realizing higher fecundity at smaller seed size, although small seeds produced late in the season will be required to overwinter before forming rosettes [38-40]. In *L. inflata*, differences in offspring traits among fruits produced at different times suggest that a transition from a high-quality to high fecundity strategy occurs as the prospect of offspring establishment diminishes through the season (i.e. from early fruit to late fruit) [39]; in our study, plants bolting at different times exhibit a similar pattern. This is likely due to the fact that seeds produced early in life are more likely to survive to reach reproductive maturity [3,5,17,36,40-42].

Bolting month was consistently the best predictor of reproductive traits. This signifies that reproductive allocation is phenotypically plastic with respect to time, and that environmental factors related to bolting month (i.e. photoperiod and/or light intensity) act as cues to trigger different allocation strategies. The consistency between lab bolting month groups which, although sharing the same photoperiod schedule, were sheltered from various stressors (e.g. wind, rain and temperature variation) experienced by their field counterparts, suggests that day length is a potent environmental cue governing allocation strategy. Because all bolting month groups were composed of the same 21 genotypic lineages, and genotype was included as an effect in our mixed model design, genotypic differences were excluded as an explanation for differences among bolting groups. Rosette size was included as an effect in our model, but did not consistently predict phenotypic differences between bolting groups, as we would have expected if plant size, or direct effects associated with plant size, largely determined reproductive allocation patterns.

Other fixed effects included in our model (environment and year), as well as interaction effects (bolting month*year, bolting month*environment, and year*environment) were significant predictors of for one or more reproductive traits, although the effect sizes (calculated for GLMs) were typically small (Tables 4-10). Differences between plant growth environments showed a consistent pattern: relative to lab-grown plants, field-grown plants generally initiated flowering at a larger size, and produced more branches and seeds, but also showed greater variability in reproductive characters. Year was not a significant predictor of most of our reproductive traits, but where it was, this was presumably due to maternal effects related to seed age (e.g. seeds germinating in 2009 were a year older than those that germinated in 2008), or, in the case of significant year*bolting month interaction effects, maternal effects that affected bolting date-specific reaction norms (i.e. size at first flowering). Easier to interpret were year*environment interaction effects, which predicted a significant amount of the variation in reproductive traits, mostly because differences in seasonal weather among years affected field plants and not lab plants. For instance, in 2008 eastern Canada experienced a warm, abnormally rainy summer, resulting in all field plants flowering at a smaller size, and producing more branches, fruit and seed. Significant bolting month*environment interaction effects showed that fluctuating weather affected plant growth at some bolting months more than others; for instance, in 2008, the pattern of increased branching became more pronounced in later bolting months, with field plants bolting in September 2008 showing the greatest number of branches produced by any block in the study. Despite the importance of these additional effects, bolting month was the only effect that significantly predicted all reproductive traits in our study.

## Conclusions

In conclusion, our data demonstrate that a classically semelparous plant exhibits variation in parity expression that is consistent with adaptive phenotypic plasticity. Other studies [13,25] have shown intriguing evidence of plasticity in reproductive life histories, and here we explicitly test whether plasticity in reproductive behaviour can be explained as plasticity in the expression of parity along a continuum. That reproductive traits vary predictably with bolting date implies that, in *L. inflata*, degree of parity responds in a plastic manner to environmental cues and its expression is continuous. This substantiates the notion that there is a meaningful continuum of reproductive traits from a pure semelparous strategy to a prolonged semelparous strategy of iteroparous-like reproductive packaging over a substantial proportion of its lifespan.

Conceptual and mathematical models identify the conditions under which annual semelparity has a selective advantage over perennial iteroparity, where semelparous and iteroparous life histories are discrete alternatives. Our data suggest that these models, because they implicitly consider invariant extreme semelparity and iteroparity, describe the special cases of endpoints of a continuum. Our results suggest that parity may be treated as phenotypically plastic and continuous over shorter time scales, as variation in key reproductive traits yields a life history that falls between the absolute extremes of pure iteroparity and semelparity. Inferences about the generality of these conclusions will require study of reproductive allocation in other classically semelparous organisms, or in iteroparous organisms in which reliable cues for residual reproductive value may be perceived by individuals.

## Methods

(i) Study species

*Lobelia inflata* (Campanulaceae) is a monocarpic plant native to Eastern North America. It has multiple flowering schedules in the wild (both annual and biennial patterns have been observed), but reproduction is always semelparous in that the plant senesces after completion of flowering. Upon germination, *L. inflata* seeds form rosettes capable of overwintering. Reproduction is initiated as a reproductive stalk forms from a mature rosette; this event is termed “bolting” and occurs predictably if size, photoperiod and light quality thresholds are exceeded [43]. *L. inflata* has perfect flowers, reproduces sexually, and is obligately self-fertilizing. Outcrossing is prevented by a stamen tube, a structure which permits the release of pollen directly onto the stigma, but does not permit the release of pollen into the air, since it is sealed [37]. Analyses of polymorphic microsatellite loci [44] have revealed no evidence that outcrossing occurs in nature.

Bolting, which marks the beginning of a transition from a vegetative to a reproductive phase, is irreversible for *L. inflata*, and thus the timing of this “decision” has important fitness consequences. Inflorescences show an acropetal flowering pattern, where flowers are produced in series from the base to the tip of the stalk (and along each branch). Each flower progresses through easily observable stages: from bud, to flower formation, to anthesis, to “inflation” (where fruits resemble small balloons–hence the name *Lobelia cinflata*”) and finally to fruit maturation. Reproduction occurs as seeds are formed inside inflated ovules; the number of seeds in a fruit has been observed to depend on environmental unpredictability and reproductive timing [39,45]. During reproduction, one or more shoots may branch off from the main stalk. Seeds disperse passively upon fruit maturation; once all fruits have reached this stage, a plant senesces.

(ii) Experimental design

Of central importance to our design was the ecological significance of the timing of bolting, marking the (irreversible and terminal) initiation of reproduction. By manipulating the date of initiation of reproduction, we were able to control the length of time that plants had to reproduce. Because reproduction is terminated relatively consistently among individuals each year (around October 15^th^) with the onset of hard frosts–a phenotypically plastic reproductive response to a range of manipulated bolting dates could be effected.

We collected seeds from dead plants at the Petawawa Research Forest (Lat. 45°99’N, Long. 77°30’W) in eastern Ontario, Canada in October 2007. To maximize the potential inclusion of a variety of genetic lineages (and preclude the possible influence of atypical genotypes), we collected seeds from 21 parent plants in the field (each at least 50 m from each other). Each seed sample was used to found an experimental population of genotypically identical replicate plants, yielding 21 (potentially distinct) genetic lineages. To obtain offspring plants from each lineage we first placed groups of 100-200 seeds on moistened filter paper in petri dishes, then germinated seeds for 10-14 days in a BioChambers SG-30 seed germinator using a 12 h /12 h day/night light regimen (at 20°C with 85% humidity).

Seedlings were then moved to individual cells (dimensions: 7.6 X 7.6 cm) within trays of autoclaved soil, and were then transferred to a Biochambers AC-40 growth chamber for prebolting rosette growth under a 16 h/8 h day/night schedule (at 24°C/18°C day/night). Trays were watered twice weekly, and a 15-5-15 liquid fertilizer mixture (200 ppm N) was added once every two weeks. Seedlings were grown for approximately 8-9 weeks, forming small rosettes. Rosettes grew undisturbed until bolting; the emergence of a reproductive stalk upon bolting may be reliably detected [37]; a stalk taller than 4 cm is diagnostic of the onset of bolting. Seed germination and seedling translocation was planned so that plants would initiate bolting at four regular intervals, targeting the 15^th^ of each month (±2 days) from June through September. Plants that bolted before the 13^th^ or after the 17^th^ of the month were excluded. The distribution of plants included in the study is shown in Table 1.

Bolted rosettes within each group were randomly assigned to one of two environments: a field site (at 45°23’N, 75°41’W) or to a growth chamber, which was programmed to mimic the photoperiod and light intensity of the field site. Lab plants were simply moved into the new chamber in their planting trays, and to minimize the difference in soil conditions between lab and field environments, field rosettes were planted along with the soil from their planting cell. Translocated plants were left to grow, reproduce and eventually senesce. Reproducing plants were monitored every two days until death.

Measurements of longest living leaf—a surrogate for rosette biomass [43], stalk height, stage of flower formation (visible bud, anthesis, mature flower, etc…), fruit maturation and branch initiation were performed on growing plants once every 4-6 days for all plants. Once they had senesced, plants were taken to the lab, measured, and harvested. At harvest, fruits were measured and removed to storage vials.

(iii) Traits measured

Seven traits were assessed in this manipulation: three phenological traits, two fruiting and two seeding traits. The three phenological traits were days to first flower, size at first flowering and flowering duration. ‘Days to first flower’ is simply the number of days between bolting and the formation of the first flower. ‘Size at first flowering’ is the distance between the base of the stalk and the pedicel of the emerging terminal flower bud (measured by Vernier caliper–error: +/- 0.01 mm); this is the plant’s height as the first flower forms. Flowering duration is the number of days between the formation of the first flower and the maturation of the last flower (as it becomes a fruit).

The two fruiting traits included branches per plant and the total number of fruits produced. “Branches per plant” was the simple count of rami protruding from the main stalk. Upon death of a plant, fruits were counted and fruit location (on branch or reproductive stalk) recorded. The “total number of fruit” produced by a plant included all fruits at all positions, both on branches and the main reproductive stalk.

The two seed traits included seed size and the total number of seeds produced. To obtain a sufficient sample size while ensuring adequate replication, seed traits were obtained from a subsample of individuals from the June and September (early and late) bolting groups. Seed size was measured by: i) sampling the ten fruits at the 100^th^/90^th^/80^th^/&c… percentile position along the raceme–all fruits were used if there were fewer than 10 in total; ii) imbibing seeds on a moistened filter paper-lined petri dish for 72 hours; and iii) measuring seed dimensions using NIHimage 1.62b7. Seed number per fruit was determined by manual count under a light microscope. Estimates of total reproductive output (the number of seeds per plant) were calculated as the product of the mean number of seeds per fruit and the number of fruits per plant.

(iv) Statistical analyses

Our aim was to assess the effect of bolting month as a predictor in a multivariate response; however, we first tested whether differences in any reproductive traits were explained by the genotype random effect. We used likelihood ratio tests to compare the proportion of total variability in response accounted for by two models [46]. The first model was a generalized linear model (GLM) that included only fixed effects and their interaction terms; and the second was a generalized linear mixed model (GLMM) that included the fixed effects and interaction effects from the first model, as well as genotypic lineage—which we considered a random effect—along with two interaction terms that included genotype. We then compared the restricted log likelihood values for each of these models to assess whether the inclusion of genotype significantly increases the predictive power of the model. This process was repeated for each of the seven reproductive traits measured. Where the GLMM did not significantly differ from the GLM in terms of the proportion of total variation explained by the model, we dropped the random effects, opting instead for the more parsimonious model. Below (Model 1 and 2) are the specifications for the two models we used.

For each of the seven reproductive traits, we constructed a GLM that included six predictors, and used a Poisson distribution and a log link. We included four fixed effects: year (categorical), bolting month (categorical), environment (i.e. lab or field–categorical), plant size (continuous, based on prebolting rosette leaf length), and two crossed effects: bolting month X year and environment X year. Effect sizes were measured using partial η^2^. Our full GLM model is below (Model 1).

### Model 1. GLM

REPRODUCTIVE TRAIT =

BOLTING MONTH + SIZE + ENVIRONMENT + YEAR + BOLTING MONTH*YEAR + ENVIRONMENT*YEAR

We also constructed a GLMM for each variable, which included: (1) all six factors from the GLM; as well as (2) “genotypic lineage”, a random effect; and (3) “genotypic lineage X environment”, “genotypic lineage X bolting month”, and “bolting month X year and environment X year”, three interaction effects. Our full GLMM model is specified below (Model 2).

### Model 2. GLMM

REPRODUCTIVE TRAIT =

BOLTING MONTH + GENOTYPE + SIZE + ENVIRONMENT + YEAR + GENOTYPE*ENVIRONMENT + GENOTYPE*BOLTING MONTH + BOLTING MONTH*YEAR + ENVIRONMENT*YEAR

This analysis contains biologically relevant random crossed effects (i.e. GENOTYPE*BOLTING MONTH), and we constructed the GLMM so that parameters were fitted using REML-based estimation, to avoid underestimation of the standard deviation of random effects [47,48].

## Availability of supporting data

The data set supporting the results of this article is available in the Data Dryad repository, DOI: 10.5061/dryad.2d218, URL: https://datadryad.org/resource/doi:10.5061/dryad.2d218[49].

## Competing interests

The authors declare that they have no competing interests.

## Authors’ contributions

PWH conducted the manipulation experiment, collected all experimental data, performed statistical analysis and participated in the drafting of this manuscript. AMS conceived of this study, performed statistical analysis and participated in the drafting of this manuscript. Both authors read and approved the final manuscript.
